# Facile Synthesis of FeCo/Fe_3_O_4_ Nanocomposite with High Wave-Absorbing Properties

**DOI:** 10.3390/ijms140714204

**Published:** 2013-07-09

**Authors:** Yu Gu, Yang Cao, Huijuan Chi, Qing Liang, Yongji Zhang, Youyi Sun

**Affiliations:** 1 Department of Mechanics, Beijing Jiaotong University, Beijing 100044, China; E-Mail: yugu@bjtu.edu.cn; 2 Research Center for Engineering Technology of Polymeric Composites of Shanxi Province, North University of China, Taiyuan 030051, China; E-Mails: 13994217058@126.com (Y.C.); huijuanchi@126.com (H.C.); liangq@163.com (Q.L.); zyj@nuc.edu.cn (Y.Z.)

**Keywords:** Fe_3_O_4_/FeCo, hydrothermal, one-step, magnetic, microwave absorbing

## Abstract

The FeCo/Fe_3_O_4_ nanocomposite was synthesized using the hydrothermal approach, in which the FeCo alloy and Fe_3_O_4_ are formed by one step. The structure of the FeCo/Fe_3_O_4_ nanocomposite was characterized by means of Scanning electron microscopy (SEM), X-ray diffraction (XRD) and X-ray energy-dispersive spectrometer spectroscopy (EDX). They show that the mass ratio of FeCo/Fe_3_O_4_ strongly depends on the reaction temperature. Such various architectures follow a stepwise growth mechanism of the composites prepared in various reaction temperatures were also discussed. It indicates that this strategy is facile, effective and controllable for the synthesis of FeCo/Fe_3_O_4_ by the one-step method. Furthermore, the magnetic and wave-absorbing properties of the nanocomposites with various structures were investigated in detail. The results show that the FeCo/Fe_3_O_4_ with higher mass ratio has higher magnetic properties. Moreover, the FeCo/Fe_3_O_4_ nanocomposite shows high wave-absorbing properties (e.g., −37.9 dB), which are expected to apply in microwave absorbing materials.

## 1. Introduction

With the objective of solving the expanded electromagnetic (EM) interference problems, considerable theoretical and experimental investigations have been focused on effective EM-wave-absorption materials with a strong absorption, a low density and a high resistivity in a wide frequency range [1,2]. Among the candidates for EM-wave absorbers, FeCo materials have high saturation magnetization, so that their complex-permeability values remain high in the gigahertz range, for which it is possible to make thinner absorbers [3,4]. However, FeCo materials have a high electric conductivity and are easy to oxidation, which drastically decreases the wave-absorbing properties [5]. Regarding this problem, among several methods for covering their surface, such as polymers, alkyl ligands, SiO_2_, metal oxides, graphitic carbon (GC) is the most suitable option from the standpoint of thermal stability, low electric conductivity and chemical inertness [6–14]. These synthesis method of core/shell nanostructures involved multi-step processes and each step was not simple, such as the synthesis of FeCo NPs with GC shells generally requires large-scale instruments for arc plasma sputtering or chemical vapor deposition (CVD) or uses the precise synthesis involving toxic Fe(CO)_5_ and a unique Co complex [12]. In addition to this, Fe_3_O_4_ have been widely applied because of their high hardness, good wear resistance, high resistivity and outstanding mechanical properties at high temperatures [15]. However, there few works reporting the preparation and wave-absorption of Fe_3_O_4_ coated FeCo particles.

In this work, a new synthesis method of Fe_3_O_4_ coated FeCo particles was reported. The present method shows an easy processing and does not high temperature treatment and multi-step to attain final FeCo/Fe_3_O_4_ nanocomposite. Moreover, the EM characteristics of Fe_3_O_4_ coated FeCo nanocapsules have been studied at 2–18 GHz. The results indicate that the Fe_3_O_4_ coated FeCo nanocapsules may be applicable in EM-wave absorptive devices.

## 2. Results and Discussion

### 2.1. Preparation of the FeCo/Fe_3_O_4_ Nanocomposite

**Figure 1:**
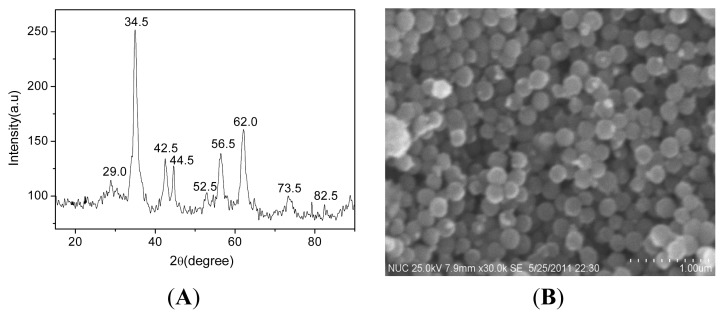
(**A**) XRD; and (**B**) SEM image of FeCo/Fe_3_O_4_ nanocomposite.

The formation of FeCo/Fe_3_O_4_ nanocomposite is firstly confirmed by the XRD and SEM as shown in Figure 1. From the Figure 1A, it is found that there are a series of characteristic peaks (at 2θ = 29.0°, 34.5°, 42.5°, 52.5°, 56.5°, 62.0° and 73.5°), which are indexed to (220), (311), (400), (422), (511), (440) and (533) of Fe_3_O_4_, respectively [15,16]. As also shown in Figure 1A, besides the characteristic diffraction peaks of Fe_3_O_4_, the diffraction peaks at 44.5° and 82.5° can be indexed to (111) and (211) of the FeCo with FCC crystal structure, respectively [17]. These results suggest that the products consist of FeCo and Fe_3_O_4_, indicating the formation of FeCo/Fe_3_O_4_ nanocomposite. The typical surface morphology of the FeCo/Fe_3_O_4_ nanocomposite is characterized by the SEM as shown in Figure 1B. It clearly shows that the large quantity of uniform spherical particles with an average diameter of 210.0 nm can be achieved via the present method. At the same time, it also shows the good dispersion of the spherical particles. These results are difficult to observe in previous works [18–20], here it is attributed to the protection of PVP on the surface of nanocomposite.

**Figure 2:**
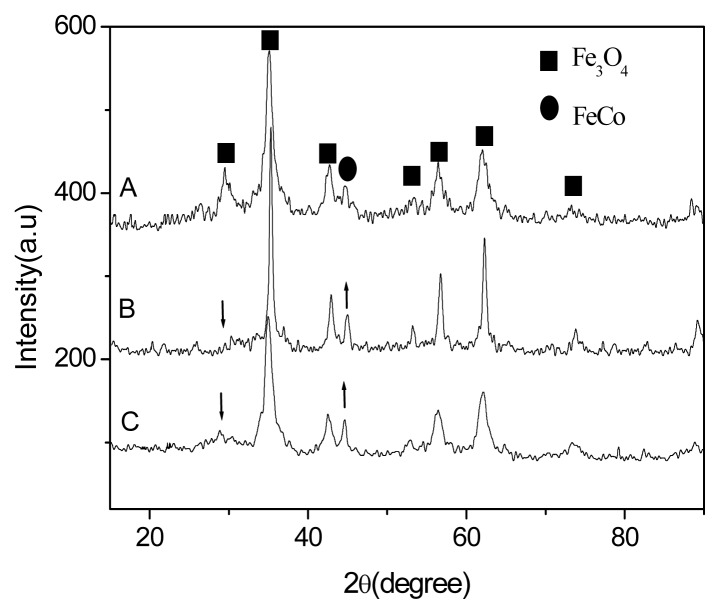
XRD pattern of FeCo-Fe_3_O_4_ composite particles prepared at various temperatures (**A**) 130 °C; (**B**) 150 °C; and (**C**) 170 °C.

It is found that the formation and structure of FeCo/Fe_3_O_4_ nanocomposite strongly depends on the reaction temperature as shown in Figure 2. In the present synthesis method, the products are difficult to be obtained at lower than 130 °C. As shown in Figure 2, all obtained products prepared at higher than 130 °C show similar distinctive diffraction peaks, which are assigned to FeCo/Fe_3_O_4_ nanocomposite. The result indicates that in order to get the single phase of FeCo/Fe_3_O_4_ nanocomposite, the reaction temperature must be higher than 130 °C. At the same time, it also shows that the intensity of diffraction peak (44.5°) assigned to FeCo (in Figure 2B,C) clearly increases compared with the result of Figure 2A. The result indicates that the mass ratio of FeCo/Fe_3_O_4_ increases with increase in reaction temperature. It is well known that the absence and presence of the core peaks in the cases of the core-shell particles depend on the thickness of the shell [21,22]. Here, the FeCo peaks in the cases of the FeCo/Fe_3_O_4_ nanocomposite are relatively slight compared with Fe_3_O_4_ peaks. These results further indicate the formation of FeCo/Fe_3_O_4_ core-shell particles and the thickness of Fe_3_O_4_ shell decreases with increase in reaction temperature.

**Figure 3:**
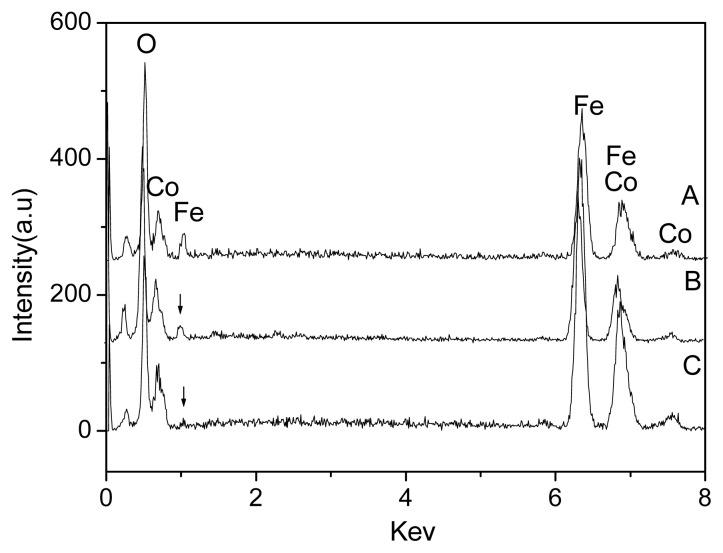
EDX spectroscopy of products prepared at various reaction temperatures (**A**) 130 °C; (**B**) 150 °C; and (**C**) 170 °C.

The above results are further confirmed by EDX spectroscopy as shown in Figure 3 and Table 1. Figure 3 shows similar curves and confirms the presence of Fe, Co and O elemental signatures for all samples [23–25]. The result further confirms the formation of FeCo/Fe_3_O_4_ nanocomposite. At the same time, the analysis also identifies that the mass ration of FeCo/Fe_3_O_4_ increases with increase in reaction temperature, as shown in Table 1. When the products were prepared at 170 °C, the largest mass ratio (*ca*. 4.8) of FeCo/Fe_3_O_4_ is observed. Here, the mass weight of FeCo is more than that of Fe_3_O_4_ in the FeCo/Fe_3_O_4_ nanocomposites. But, according to results of Figure 2, the FeCo peaks are difficult to observe, and the Fe_3_O_4_ peaks are clear and strong. The result is attributed to that the surface of FeCo is coated with Fe_3_O_4_, indicating the formation of FeCo/Fe_3_O_4_ core-shell particles [21,22,25].

**Figure 4:**
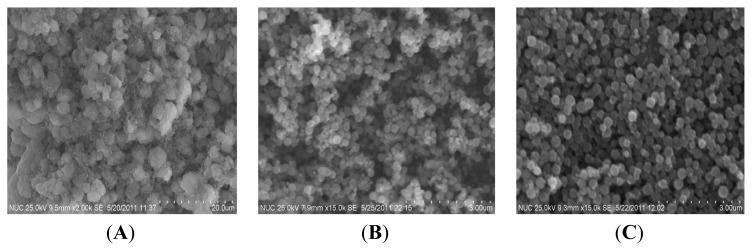
SEM images of products prepared at various reaction temperatures (**A**) 130 °C; (**B**) 150 °C; and (**C**) 170 °C.

The surface morphology of the FeCo/Fe_3_O_4_ nanocomposite as function of reaction temperature was also characterized by SEM images as shown in Figure 4. It shows that the products prepared at 130 °C exhibit irregular morphology. When the reaction temperature is higher than 130 °C, the irregular particles transferees into spherical particles as shown in Figure 4B,C. At the same time, it also shows that the average size of spherical particles increase from 210.0 to 320.0 nm with increase in reaction temperature from 150 to 170 °C. It is well known that the formation of small particles is kinetically favored and that the formation of large particles is thermodynamically favored. From a standpoint of kinetics, small particles are easier to nucleate. However, small particles have larger surface area-to-volume ratio and represent a higher energy state than large particles. Hence, small FeCo/Fe_3_O_4_ nanocomposites are easily formed at lower temperature.

**Scheme 1:**
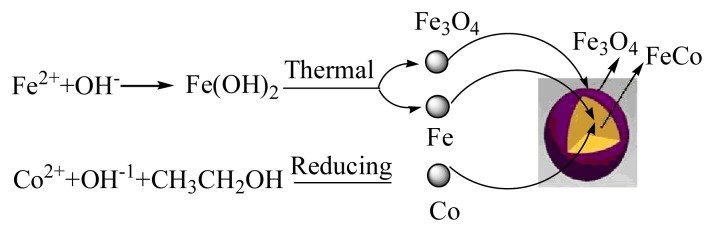
Illustration of formation of core-shell composite particles by one-step method.

On the basis of the above experimental results, it is believed that the FeCo/Fe_3_O_4_ nanocomposite is prepared by a one-step method. Then what is the possible formation process for the FeCo/Fe_3_O_4_ composite particles? It is well known that as iron is a metal much more electropositive than cobalt, the reduction of Fe° cannot be achieved by polyols which are too weak reducing agents. Generally, the reducing agent (e.g., NaBH_4_ and N_2_H_4_·H_2_O) was introduced into the reaction, and the FeCo alloy was prepared by the hydrothermal approach [19–21]. Of course, the FeCo/Fe_3_O_4_ is difficult to be formed in these works [26–28]. Here, an alternative way to try to produce Fe° particles by precipitation from a liquid phase is the disproportionation of ferrous hydroxide. Actually, the formation of iron from suspension of Fe(OH)_2_ has been observed in water at high temperature [29], at the same time, the Fe_3_O_4_ is also easily formed as shown in Scheme 1. In particular, the increasing reaction temperature during the precipitation of the starting hydroxide increases Fe° formation yield [28]. In addition to this, the solid hydroxides Co(OH)_2_ initially form when excessive NaOH solution is added to the mixture solution of Co(Ac)_2_·4H_2_O. The Co^2+^ ions also released from their solid hydroxides are simultaneously reduced to metallic atoms, which spontaneously form FeCo alloyed nuclei. The relevant growth mechanism of composites can be summarized as shown in Scheme 1.

### 2.2. Properties of the FeCo/Fe_3_O_4_ Nanocomposite

**Figure 5:**
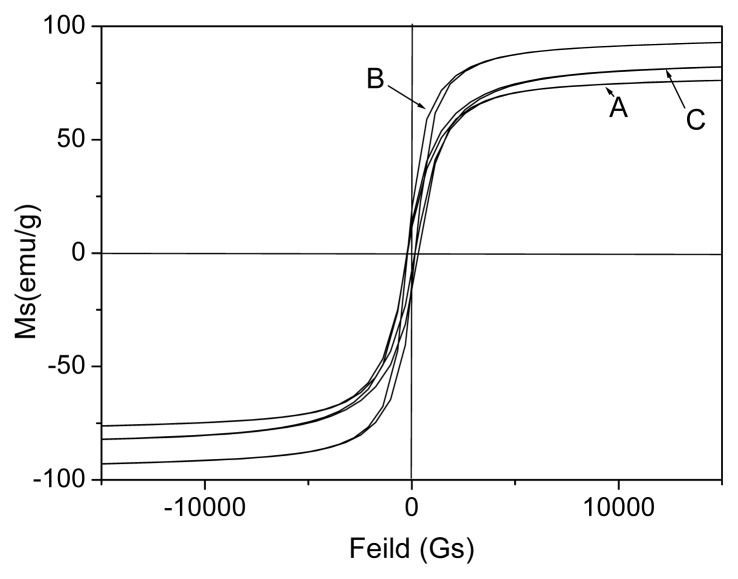
VSM of products prepared at various reaction temperatures (**A**) 130 °C; (**B**) 150 °C; and (**C**) 170 °C.

The M-H loops of products prepared at various temperatures were further compared as shown in Figure 5. It clearly shows that the Ms of nanocomposite increases with increase in reaction temperature as shown in Table 2. The highest *Ms* (*ca*. 112.8 emu/g) of nanocomposite prepared at 170 °C is observed in this study. These results are attributed to that the mass ratio of FeCo/Fe_3_O_4_ increases with increases in reaction temperature, which is consistent with the result of XRD. Moreover, it is found that the *Ms* of the nanocomposite is almost similar with that of pure FeCo alloy [26–28]. The result indicates that the Fe_3_O_4_ coated FeCo effectively shields the magnetic nanoparticles (FeCo alloy) against environmental degradation, and keeps the magnetic properties unchanged. In addition to this, it shows that the effect of reaction temperature on the coercivity (*H*c) is slight.

**Figure 6:**
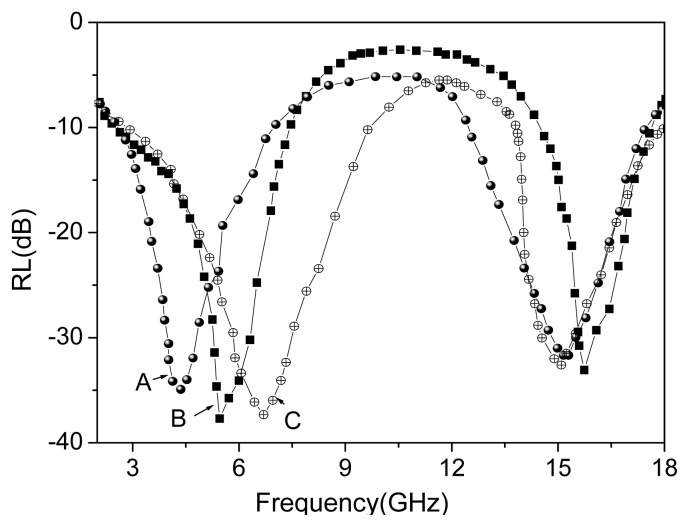
Reflection losses of products prepared at various reaction temperatures (**A**) 130 °C; (**B**) 150 °C; and (**C**) 170 °C.

The reflection loss (RL) values of the FeCo/Fe_3_O_4_ nanocomposites as a function of frequency are shown in Figure 6. It clearly shows that an optimal RL of −37.9 dB is reached at 5.2 GHz and the absorption exceeding −20.0 dB is obtained in the range (4.9–8.6 GHz and 14.0–16.8 GHz). Compared with FeCo alloy, the maximum RL of FeCo/Fe_3_O_4_ nanocomposites increases by about 20%–40% [30–32]. The special core-shell structure of the FeCo/Fe_3_O_4_ nanocomposites may additionally contribute to the excellent absorption properties as shown in following: (1) the dielectric Fe_3_O_4_ shell is responsible for increasing the dielectric losses in the FeCo nanocapsules; (2) as a magnetically inactive layer, it causes a demagnetizing field and prevents the magnetic interaction between the magnetic components, which leads to increased magnetic losses in the FeCo nanocapules. These features illuminate that the microwave absorbing properties of this composite are mainly due to the magnetic and dielectric loss together. At the same reason, the Fe_3_O_4_ shell in the FeCo/Fe_3_O_4_ nanocomposites is thicker, and the FeCo/Fe_3_O_4_ nanocomposites are wider absorbing-bandwidth as shown in Figure 6. Compared to expensive microwave absorbing materials, the price of these magnetic spheres is very low; therefore, this is an effective way to reduce the cost of absorbing materials and broaden absorbing bandwidth by adjust the preparation conditions of FeCo/Fe_3_O_4_ nanocomposite.

## 3. Experimental Sections

### 3.1. Materials

The chemicals used in the experiments were ferrum (II) chloride (FeCl_2_·4H_2_O), Cobalt acetate (Co(Ac)_2_·4H_2_O), sodium hydroxide (NaOH), polyvinyl pyrrolidone (PVP), ethanol and ethanediol. All chemicals were purchased from Shanghai Chemical Reagents Company and used without further purification.

### 3.2. Preparation of FeCo/Fe_3_O_4_ Nanocomposite

In a typical procedure, 0.58 g FeCl_2_·4H_2_O and 0.31 g Co(Ac)_2_·4H_2_O were dissolved in 30 mL ethanediol solution. Subsequently, 10 mL ethanol containing 0.05 g PVP and 1.6 g NaOH was added into above solution. The mixture was stirred vigorously and then was transferred into a Teflon cup in a stainless steel-lined autoclave. The autoclave was maintained at 130 °C (150 °C or 170 °C) and then was cooled down to room temperature. A black fluffy solid product was deposited on the bottom of the Teflon cup, indicating the formation of FeCo/Fe_3_O_4_ nanocomposite. The final product was collected by a magnet and rinsed with distilled water and ethanol three times to remove any salts, and then dried in a vacuum oven at 40 °C for 6 h.

### 3.3. Characterization

The X-ray diffraction (XRD) patterns of the samples were recorded with a Rigaku D/Max-2000 diffractometer equipped with a Cu KR radiation source (λ = 0.15, 418 nm). The scanning range was from 15° to 90° and the scanning interval was 0.01°.

Morphologies of the samples were studied by a Hitachi Su-1500 scanning electron microscope (SEM). The element composition was characterized by a Horiba EX-250 X-ray energy-dispersive spectrometer (EDX) associated with SEM.

The hysteresis loops were conducted by using a Model-4HF vibrating sample magnetometer at room temperature with a maximum magnetic field of 15 kOe. For magnetization measurements, the powder was pressed strongly and fixed in a small cylindrical plastic box.

The microwave-absorbing properties were measured on an Agilent Vector Network Analyzer in the 2–18 GHz, composite materials were prepared by dispersing the FeCo/Fe_3_O_4_ nanocomposite in paraffin wax with cylindrical toroidal specimens of 2 mm thickness, respectively, and the mass fraction of powders was 50%.

## 4. Conclusions

The FeCo/Fe_3_O_4_ nanocomposite was synthesized by using one-step solution method. Their structure and magnetic properties were determined by the XRD, SEM and VSM. These results indicate that the formation and magnetic properties of the composite particles depend on the reaction temperature. In order to obtain FeCo/Fe_3_O_4_ composite particles with uniform size and shape, the reaction temperature should to be higher than 130 °C. The high Ms (112.8 emu/g) of composite particles prepared at 170 °C was obtained. Moreover, the FeCo microspheres exhibit excellent EM properties. It is found that the optimal value of RL is −37.9 dB at 5.4 GHz for a layer 2.0 mm layer. The present work provides a new synthesis method of FeX (Co, Ni and so on)/Fe_3_O_4_ composite particles, which is expected to apply in EM materials.

## Figures and Tables

**Table 1:** Element weight of products prepared at various reaction temperatures (**A**) 130 °C; (**B**) 150 °C; and (**C**) 170 °C.

Samples	A			B			C		
Element	Co	Fe	O	Co	Fe	O	Co	Fe	*O*
Mass Weight (%)	30.1	51.2	8.5	36.3	51.3	6.3	39.9	49.6	*4.3*
Mass ratio	1.9			3.1			4.8		

**Table 2:** Ms of products prepared at various reaction temperature.

Reaction conditions	130 °C	150 °C	170 °C
Ms (emu/g)	88.6	92.4	112.8
Hc (Gs)	148.5	148.0	148.2
